# Neural correlates of facial expression processing during a detection task: An ERP study

**DOI:** 10.1371/journal.pone.0174016

**Published:** 2017-03-28

**Authors:** Luxi Sun, Jie Ren, Weijie He

**Affiliations:** 1 School of Economics and Business Administration, Chongqing University, Chongqing, China; 2 Laboratory of Emotion and Mental Health, Chongqing University of Arts and Sciences, Chongqing, China; Liaoning Normal University, CHINA

## Abstract

Given finite attentional resources, how emotional aspects of stimuli are processed automatically is controversial. Present study examined the time-course for automatic processing of facial expression by assessing N170, and late positive potentials (LPPs) of event-related potentials (ERPs) using a modified rapid serial visual presentation (RSVP) paradigm. Observers were required to confirm a certain house image and to detect whether a face image was presented at the end of a series of pictures. There were no significant main effects on emotional type for P1 amplitudes, whereas happy and fearful expressions elicited larger N170 amplitudes than neutral expressions. Significantly different LPP amplitudes were elicited depending on the type of emotional facial expressions (fear > happy > neutral). These results indicated that threatening priority was absent but discrimination of expressive vs. neutral faces occurred in implicit emotional tasks, at approximately 250 ms post-stimulus. Moreover, the three types of expressions were discriminated during the later stages of processing. Encoding emotional information of faces can be automated to a relatively higher degree, when attentional resources are mostly allocated to superficial analyzing.

## Introduction

Facial expression processing occurs during many situations, and people are both highly efficient and fast at identifying emotional information from others’ expressions [1]. Furthermore, this processing also occurs when emotion-related content is not necessary. Input expression signals elicit unintentional responses; hence, processing results in automatic extraction of emotional information [2–4].

Electrophysiological data reveal that visual evoked potentials are sensitive to the emotional content of facial expressions at very early stages of processing [5, 6]. Additionally, emotional stimuli, compared with neutral stimuli, elicit greater visual cortex activation during passive viewing [7, 8]. This activation is associated with “emotional attention” which is defined as a predisposition to spontaneously collect all processing resources for emotional information [9, 10].

Rellecke and colleagues [11] used a face-words discrimination task to determine that processing emotionality in faces, not words, can induce activity indexed by early posterior negativity (EPN). This group also assessed the automaticity of facial expression processing by systematically comparing ERP differences elicited by three kinds of faces under various depths of processing conditions [12]. They found that automatic enhanced encoding of angry faces were indicated by P1, N170, and EPN in the early processing stages; however, happy expressions that occurred later depended on participant intentions. These findings contribute to understanding of cognitive processing of expressions.

However, we aimed to create experimental conditions that minimize intentional observer allocation of attentional resources to each expression because expressions are usually perceived rapidly and unconsciously. Therefore, we designed an experimental task requiring rapid face detection; a target stimulus is briefly presented as a component of a stimuli stream and observers determine only whether it is presented, thereby enabling more automatic access to the expressional contents of the target face. A rapid serial visual presentation (RSVP) paradigm met our criteria, as stimuli are presented sequentially and rapidly (approximately 100 ms/item). When the interval between two targets is short (200–500 ms), detection of the second target is impaired by the first target and this phenomenon is called Attentional Blink [13, 14]. During the period of attentional blink, processing resources are not adequate to recognize all facial expression information.

Facial expression categorization has been assessed [15] using an RSVP paradigm with three processing stages: fear popup, emotional/non-emotional discrimination, and complete separation. A subsequent study depicted similar phases in emotional adjective processing [16], and both studies indicated that emotion processing is spontaneous, is not significantly affected by competing stimuli, and does not require attentional resources.

The current study extended previous findings by testing whether the RSVP model requires emotion categorization, in healthy participants. The present study differs from previous ones because observers were instructed to detect whether an upright face appeared in the stimuli sequence. ERP amplitudes N1, P1, N170, vertex positive potential, N3, and P3 have been used in previous experiments to reveal periodic activities. In the present study, we used a simplified method to index the activities in decoding facial expression information; only P1, N170, and LPP were analyzed. The reasons for inclusion of these components include that P1 can be distinguished from N1 because N1 is more sensitive to attentional manipulations demanding feature discrimination rather than detection [17, 18]. N170 and VPP are dipoles for each other, so we choose only N170 represented for the next processing stage. Additionally, LPP reflects brain electrical activity during both automatic and controlled attentional processing for emotional information.

P1 is a positive-direction ERP component that primarily occurs in the bilateral occipital areas, with onset latency between 60 and 90 ms, and peaks at approximately 100–130 ms post-stimulus. Furthermore, there is an early effect of facial expressions on distinctive P1 responses for fearful compared to neutral faces [2, 19, 20]. This effect is interpreted as related to processing the coarse features of stimuli. However, magnetoencephalography and electrophysiological evidence have demonstrated that P1 is affected by facial emotion, providing evidence for rapid facial emotion processing [5, 21, 22].

N170 is a negative amplitude component detected at 120–220 ms, and peaks at approximately 170 ms post-stimulus in the lateral occipito-temporal electrode. N170 components elicited from the right hemisphere have larger amplitudes than those from the left hemisphere [23]. The main feature of this electro-physiological component is greater responses to faces than other kinds of stimuli [24, 25]. Some studies have found that facial expressions regulate and control activity indexed by N170 [2, 5].

LPPs consist of a sustained positivity that occurs at approximately 400–600 ms following stimulus onset, which increases more for emotional compared to neutral images [26, 27]. Since these stimuli automatically captivate attention and are preferentially processed by the brain, LPPs are considered indexes of motivated attention [28, 29]. Furthermore, LPPs indicate more elaborate emotion-related processing such as conscious evaluation [30] and perceptual analysis [31], and are also related to high-level recognition processing. For example, Langeslag and colleagues [32] proposed that LPPs are related to an individual’s experiences, as they were larger in response to the observer’s beloved person compared to a friend or an unfamiliar person. However, electro-cortical responses to emotional stimuli were not influenced by task difficulty [29], thereby providing evidence that emotional stimuli automatically receive attentional processing resources. Furthermore, LPPs may also be related to a rapid and dynamic course of attention to emotional stimuli [33].

The automaticity of emotional processing has often been examined by regulating concurrent multiple task demands. Erk, Abler, and Walter [34] reported that a demanding and distracting task decreased neural activity related to emotional involvement; however, it did not affect neural activity related to actual emotional stimuli processing, indicating that participants could process emotional stimuli to some extent while distracted. Therefore, in the current study, we hypothesized that if the detecting of different facial expression showed different spontaneous process, not affected by a competitive target and not requiring attentional resource, then it should be classified as an automatic process. Participants would be able to automatically process more information than what were required according to instructions that we provided before the experiments. This automatic process may have highly efficient advantage in acquiring valuable knowledge for judging emotional content.

## Materials and methods

### Participants

Sixteen undergraduates (8 men and 8 women; 19–24 years old) participated in our experiment; none dropped out of the experiment once it began. All of them were right-handed, had normal or corrected-to-normal vision and reported no history of neurological diseases and no structural brain abnormality. All participants were provided written informed consent prior to the study. The study was approved by Chongqing University Human Research Institutional Review Board in accordance with the Declaration of Helsinki (1991).

### Stimuli

Experimental materials comprised 30 human facial expression images and 3 upright house images as visual stimuli. We picked out the face pictures from the native Chinese Facial Affective Picture System (CFAPS) to generate emotional stimuli, with 18 different images of normal faces (6 happy, 6 neutral and 6 fearful, evenly divided between male and female) as targets and 12 of inverted neutral counterparts as distractions. Upright face pictures were chosen in such a manner that varied prominently in valence from one to another *F*_*2*,*15*_ = *338*.*03*, *p < 0*.*001* (*M ± SD*, *happy*: *6*.*90 ± 0*.*22*, *neutral*: *4*.*70 ± 0*.*31*, *fearful*: *2*.*75 ± 0*.*29*) but were approximate in arousal *F*_*2*,*15*_ = *0*.*66*, *p > 0*.*05* (*happy*: *5*.*55 ± 0*.*40*, *neutral*: *5*.*28 ± 0*.*44*, *fearful*: *5*.*28 ± 0*.*57*). Males’ and females’ face appeared equally in the pictures sequence. They were similar to each other in size, background, spatial frequency, contrast grade, brightness and other physical properties. All faces were ruled out the hair, moustache so that merely interior characteristics of them were kept. We managed to cut every face into the shape of an oval using Adobe Photoshop CS4 software. Each stimulus subtended 5.7 × 4.6° of viewing angle, and the screen resolution was 72 pixels per inch. All stimuli were displayed in the center of the screen.

### Procedure

**Fig 1:**
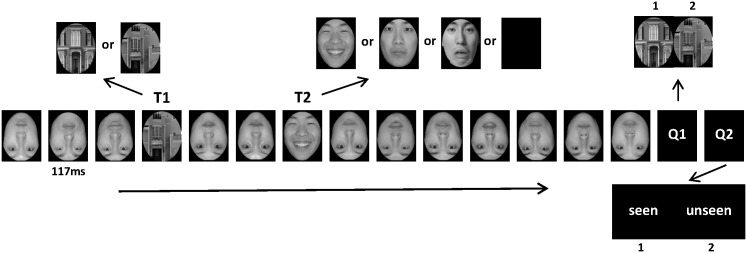
Experimental procedure and stimuli illustration: Participants were required to make effort on deciding which house were represented and whether a hairless face appeared or not by pressing ‘1(house of one type and presented face)’or ‘2 (house of other type and absent face).

Participants were seated at a viewing distance of 70 cm in front of a computer screen in a dimly lit, sound attenuated room. Experiment started with a white and a blue fixation point lasting for 500 and 300 ms presented sequentially and separately in the center of the screen. After a short while, there would be 14 images of distracting and target stimuli, with each being kept only 117 ms. Distractive stimuli comprised 12 inverted neutral faces. T1 and T2 were chosen randomly from three upright images of a house and three kinds of face with equal possibility, respectively. In particular, each condition of T2 (happy, fearful, neutral, and absent) would appear with the same possibility of 25%. T1 appeared in the third position in the stimuli series. T2 emerged after two distractive images after T1. Subjects were required to respond to one or two questions after the presentation of these pictures in previous experiment [15, 16]. In this study, they needed to reply both of these questions. By pressing one of three buttons of the response box with their right hand, the first task was to make a judgment which house they saw just now. The second task was to report whether they had seen a face or not as we told them before. Observer did not have to point out the specific expression types (Fig 1). Still, this judgment should be made as accurately as possible and there was no time limit for subjects to confirm their responses.

Inter-stimulus interval (ISI) lasted for 500 ms, during which the screen kept black and blank. The whole experiments were divided into 3 identical blocks. The observer was permitted to have a short rest between the two consecutive blocks. Each subject completed a total of 240 trials. The study includes four conditions (three facial expressions and ‘T2 absent’) and 60 trials were presented for each condition. The whole experimental procedure was performed on E-Prime 1.2.

### ERP recording

Scalp electrical activity (EEG) was recorded with tin-electrodes mounted in a 64-electrode elastic cap (Brain Product), with the initial reference on the bilateral mastoids. Vertical and horizontal electro-oculogram (EOG) was measured by additional electrodes placed around the eye. All inter-electrode impedance was kept below 5 kΩ throughout recording. The electroencephalogram (EEG) and EOG were amplified using a 0.01–100 Hz band-pass filter and continuously sampled at 500 Hz/channel. Trials with EOG artifacts (mean EOG voltage exceeding ± 80 μV) and those contaminated with artifacts because of amplifier clipping, where peak-to-peak deflection exceeding ± 80 μV, were cut off from averaging.

### Data measure and analysis

We used one-way repeated ANOVAs to compare the response accuracy of the factor emotional type (three levels: happy, neutral, and fear). Then mean ERP amplitudes were recalculated to average reference and analyzed, for their epochs were generated off-line, beginning 200 ms prior to T2 stimuli onset and lasting for 1200 ms. What counted, trials were accepted only when subjects correctly responded to both T1 and T2. But only T2 was the analyzed target stimulus.

In the present study, P1, N170, LPP components were measured and analyzed the amplitudes as extended on the topography and previous findings [12, 35]. These components were measured and analyzed by the amplitude (P1, N170). The following 9 electrode sites (Pz, P3, P4, POz, PO3, PO4, Oz, O1 and O2) were selected for statistical analysis of the P1 component (140–220 ms); And 4 electrode sites (P7, P8, PO7 and PO8) for N170 (220–320 ms). The mean amplitudes of LPP component (400–500 ms) was calculated at the 21 electrode sites (Fz, F3, F4, FCz, FC3, FC4, Cz, C3, C4, CPz, CP3, CP4, Pz, P3, P4, POz, PO3, PO4, Oz, O1 and O2). A three-way repeated measures analyses of variances (ANOVAs) on the amplitude of P1, N170 and LPP component was conducted with expressions, hemispheres (left, medial and right for P1 and LPP; left and right for N170) and electrodes (refer to previous electrode sites mentioned above) as within-subjects factors. These effects with two or more freedom were adjusted for violations of sphericity according to the Greenhouse-Geisser correction.

## Results

### Behavioral performance

The result showed that marginal significant main effect at emotional types (*F*_*2*,*30*_ = *2*.*94*, *p = 0*.*083*, *η2 p = 0*.*164*). The pairwise comparison indicated that happiness expressions (*94*.*61 ± 4*.*71%*) elicited marginal significant higher accuracy than did neutral faces (*92*.*02 ± 4*.*52%*, *p = 0*.*077*), but fear (*94*.*07 ± 5*.*50%*) and happy (*p = 1*.*000*), fear and neutral faces (*p = 0*.*474*) did not show significant accuracy differences.

### ERP data analysis

#### P1

P1 amplitudes showed significant main effect at hemispheres (*F*_*2*,*30*_ = *5*.*65*, *p = 0*.*011*, *η2 p = 0*.*273*) and electrodes (*F*_*2*,*30*_ = *22*.*29*, *p < 0*.*001*, *η2 p = 0*.*598*). Right hemisphere (*2*.*66 μV*) elicited larger P1 amplitudes than medial (*1*.*79 μV*, *p = 0*.*004*), while left hemisphere (*2*.*07 μV*) did not show any significant amplitude differences with medial (*p = 0*.*938*) and right hemisphere (*p = 0*.*196*). O1/z/2 electrodes (*3*.*08 μV*) elicited largest P1 amplitudes than PO1/z/2 (*2*.*31 μV*, *P = 0*.*022*) and P1/z/2 (*1*.*14 μV*, *p = 0*.*001*) electrodes, PO1/z/2 (*2*.*31 μV*, *p = 0*.*022*) electrodes elicited larger P1 amplitudes than P1/z/2 electrodes (*p < 0*.*001*). While P1 amplitudes did not show significant main effect at emotional types (*F*_*2*,*30*_ = *0*.*86*, *p = 0*.*432*, *η2 p = 0*.*054; happiness 2*.*03 μV*, *neutral 2*.*19 μV*, *fear 2*.*30 μV*).

#### N170

**Fig 2:**
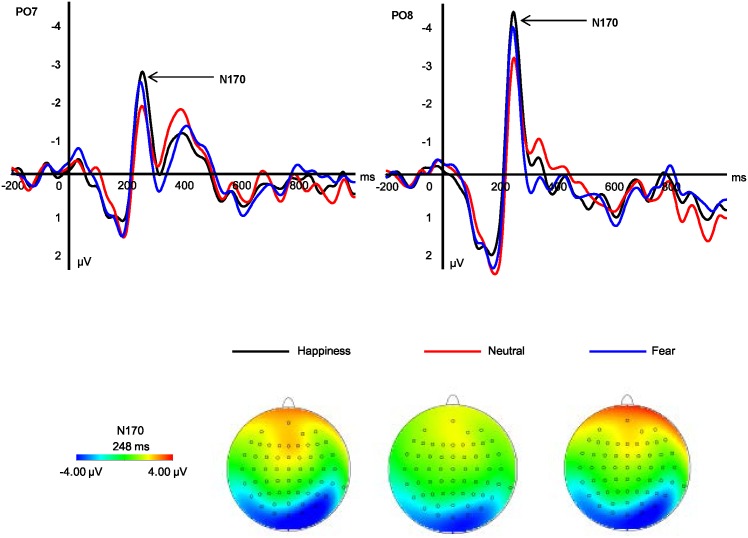
Grand average ERP component of N170 at the indicated sites and corresponding topography for each condition.

N170 amplitudes showed significant main effect at emotional types and hemispheres (*F*_*2*,*30*_ = *11*.*13*, *p < 0*.*001*, *η2 p = 0*.*426; F*_*1*,*15*_ = *3*.*52*, *p = 0*.*080*, *η2 p = 0*.*190*). The pairwise comparison indicated that right hemisphere (*-4*.*25 μV*) elicited larger N170 amplitudes than left hemisphere (*-3*.*10 μV*, Fig 2). The happy expressions (*-4*.*09 μV*, *p = 0*.*002*) and fearful expressions (*-3*.*85 μV*, *p = 0*.*010*) elicited larger N170 amplitudes than neutral expressions (*-3*.*09 μV*), while the former two emotional conditions did not show any significant amplitude differences (*p = 0*.*758*).

#### LPP

**Fig 3:**
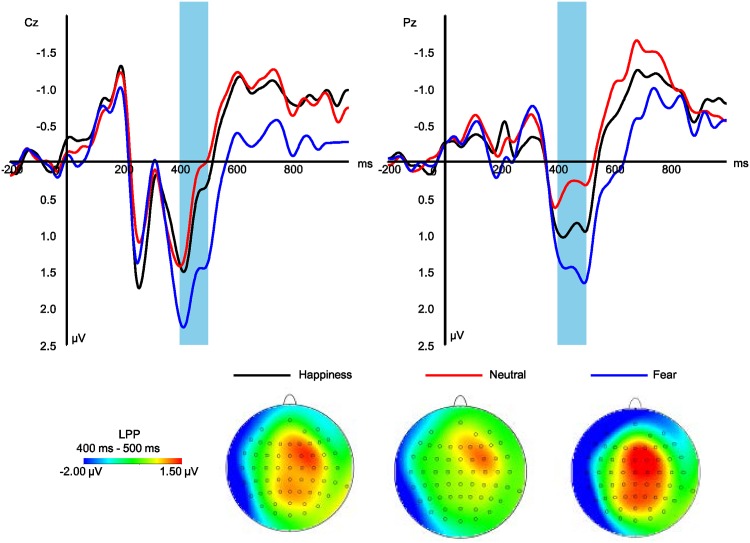
Grand average ERP component of LPP at the indicated sites and corresponding topography for each condition.

**Fig 4:**
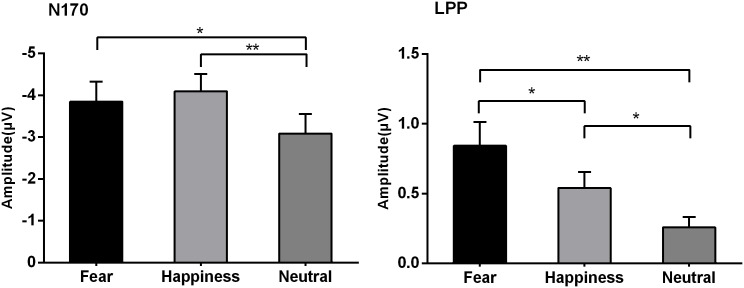
N170 and LPP amplitudes during the corresponding time window for 3 types of emotion. *, *p* < 0.05; **, *p* < 0.01. Y axis, amplitude (μV, mean ± SE).

LPP amplitudes showed significant main effect at emotional types and hemispheres (*F*_*2*,*30*_ = *11*.*17*, *p = 0*.*002*, *η2 p = 0*.*427; F*_*2*,*30*_ = *3*.*55*, *p = 0*.*043*, *η2 p = 0*.*191*). The pairwise comparison indicated that fearful expressions (*0*.*84 μV*, Figs 3 and 4) elicited larger LPP amplitudes than did happy (*0*.*54 μV*, *p = 0*.*047*) and neutral expressions (*0*.*26 μV*, *p = 0*.*007*), and happy ones showed larger LPP amplitudes than did neutral ones (*p = 0*.*017*). Medial sites (*0*.*71 μV*) elicited larger LPP amplitudes than left hemisphere (*0*.*18 μV*, *p = 0*.*056*).

## Discussion

Response accuracy was better for happy-face stimuli than for neutral-face stimuli; however, there were no significant differences among other conditions. This finding suggests that happy facial expressions are more easily perceived from a stream of rapid stimuli presentations, which is inconsistent with the results of previous studies [15]. Happy facial expressions showed a processing advantage [36] in the current study, compared to fearful facial expressions. Happy faces themselves have different low-level physical properties compared to the other two kinds of expressions, which may explain advantages of processing this type of facial expressions when distinguishing the expression type is not required. No other differences were observed in the presence of the attentional blink period, including threatening stimuli preference effects. Differences in accuracy between any two of these types of facial expressions were not significant, and therefore do not reflect internal processing.

Consistent with previous findings [15, 16], our findings support an automatic three-stage model of perceiving facial expressions. The enhanced processing was observed at the first stage of processing, reflecting rapid neural processing of the target face. However, rapid and distinct perception of fearful facial expressions did not occur soon after the target onset. The latter two stages were similar to the corresponding stages; emotional expressions are preferentially processed later, in the second stage. Finally, all expressions are differentiated in the third stage.

Earlier investigations indicated that compared to positive or neutral stimuli, responses to negative stimuli were enhanced, indicating a negativity bias in attention allocation (For example: [19]). This process showed characteristics including speediness, coarseness, and automation. Especially for processing of fearful expressions, larger P1 amplitude effects were found regardless of spatial locations [5], intentional states [12], and visual cues [37]. In an affective priming task [38], larger occipital P1 potentials occurred in response to fearful faces compared to happy faces, indicating that recognition of ambiguous faces (such as a surprised face) is susceptible to threat information for visual stimuli, which is represented by millisecond-based differences in responses. However, our results showed that this difference was not appeared during a rapid sequential stimuli stream.

P1’s effect of encoding fearful expressions was absent in current study. We reconsidered the difference between their experimental designs and took it as the main reason. For previous one, we demanded participants to make a judgment of emotional type of the presented facial stimulus. The procedure needs elaborated analysis in order to extract relevant information [12]. While detecting whether a facial stimuli was presented consumed only superficial analysis in current study. This procedure conveyed some kind of default mode for processing emotional stimuli unconstrained by any additional task-imposed requirements. They differ in depth of processing, and what’s more, their processing can be classified as two types of intentional states, one explicit and one implicit [39]. This result would argue that in competitive resources surroundings, and human might ignore detailed information at very early stages for perceiving new coming faces. At the very least, emotion encoding was not pronounced.

Vuilleumier and Pourtois [40] demonstrated that the P1 advantage effect for fearful expressions results from processing of low-level visual features. The absence of P1 amplitudes for emotional expressions may indicate that top-down effects in visual system searching for threatening cues are also based upon bottom-up mechanism; intentionally, participants did not allocate adequate attentional resources. As predicted, during the attentional blink period, limited attentional resources considerably modified actual performance of threat perception.

But, an overt second stage was found in accordance with previous processing expression studies [15, 35]. The N170 component may reflect processing at this stage. Fearful and happy expressions represent two types of typical emotional expressions. The larger N170 amplitudes elicited by emotional expressions [35, 41] suggested that they were sensitive to general emotional facial expressions; however, they could not distinguish specific expressions, and it showed an emotional preference and such facial processing may occur in or near the fusiform gyrus [15, 42].

A more automatic expression-processing task produced similar results, suggesting that neural processes related to distinguishing emotional faces from non-emotional faces may be similar to that in our previous study. For present one, we chose lag2 to focus on the processing during attentional blink period. And results are consistent with those of many discrimination tasks [43, 44]. Though processing fearful faces is advantageous in conditions with limited attentional resources, fearful faces may not attract human attention when distinguishing faces is not needed; the details of facial expression may be ignored or processed later. Convincing results may be determined in the next stage of processing.

Processing emotional facial expressions reflected by LPPs showed different in the third stage. Previous studies have also demonstrated that LPP amplitudes reflect the degree of essentialness for affective stimuli, which elicit larger LPP amplitudes compared to neutral stimuli [45, 46]. Enhanced P300 (280–450 ms) amplitude occurs for fearful facial expressions [35], suggesting that signals containing potential danger can enhance elaborate processing of stimulus and context. Since the final decision regarding distinguishing the expressions was not a component of our instructions, it is not known whether participants completed the procedure. However, the participants indeed performed some intentional categorization by result of their neural activity. Because in present results, face processing related potentials (LPPs) increased in the parietal regions, and previous ERP studies [47] demonstrated that it reflected further evaluation of information related to the affective valence of a face and this stage of processing expressions depends on observers’ own intentions.

In contrast, LPP is a very sensitive index of attention manipulations related to modulating a limited number of resources [48]. Participants quickly focused their attention on the task-relevant stimuli, rather than on other inverted distractors. The differences between the LPP amplitudes indicated an elaborate processing stage for emotional meanings. Moreover, LPPs reflect the gateway to conscious processing [30, 49]. Considering our findings, facial expression information indexed by enhanced LPPs may be linked to distinct representations in working memory during this stage, which is consistent with previous studies (For example: [4]). Enhanced LPPs may also reflect that participants recognize intrinsic meaning from the distinct face representations. Therefore, the detection task in our RSVP paradigm may have induced coarse processing of the stimuli, and may also automatically produce meaningful learning regarding the meaning conveyed in the faces.

In the present study, the peak latencies of P1 and N170 were approximately 180 and 270 ms, respectively, which are 50 ms and 100 ms later than those typically reported [50]. The reasons for these differences have been discussed in previous studies [15, 16], and this designation is based on the scalp distribution of the two components, which were consistent with the expected parieto-occipital regions (P1) and occipito-temporal regions (N170). In particular, N1 was recorded at the same scalp electrode points as in a previous study. Therefore, results of N170 component can be directly compared with the results from a different task in previous study [15]. Furthermore, the time delays used for P1 and N170 amplitudes are likely attributed to the RSVP paradigm in this experiment, as completing two tasks during a rapidly presented stimuli stream is a relatively difficult task for participants. Both of the delays to the target stimulus are therefore minimal.

P1 amplitudes elicited in the parietal and parietal-occipital regions of the right hemisphere were greater than those elicited in the left hemisphere. The present data indicated right hemisphere dominance for the N170 amplitude, which is consistent with increased N170 over the right hemisphere observed for both real and schematic faces [51]. Previous studies have shown that the right hemisphere may be preferentially involved in processing that occurs later than 200 ms after stimulus onset [52, 53].

Finally, one may expect a limitation that enhanced processing at the neural level associated with improved performance as indicated by ERP components (P1, N170, LPP) should be reconciled with the finding of increased accuracy. However, increased neural processing of emotional expressions is not necessarily related with improved performance [54]. Automatic processing at the neural level may be based on the stimuli’s affective relevance ensuring behavioral adaptability in the real world. But more research is needed for accounting for the disparity between behavioral and neural activity

## Conclusions

We tested the automaticity of facial expression processing when no intentional categorization task was required. Therefore, our procedure reflected relatively automatic facial expression encoding. The amplitudes of P1, N170, and LPP components supported three stages of facial expression processing. There was no processing preference for fearful faces, and early threatening information appeared to be ignored. However, our findings still support an automatic multi-stage processing of facial expressions, for participants automatically distinguished between the emotional expressions and non-emotional expressions, and automatically explore the specific features of three types of facial expression later after the stimuli onset. We examined automatic facial expressions processing in the relative limited attentional resources surroundings. That may build a foundation for considering how to improve humans’ perceiving outside world in an automatic procedure.
